# Enhancer RNAs in transcriptional regulation: recent insights

**DOI:** 10.3389/fcell.2023.1205540

**Published:** 2023-05-17

**Authors:** Qi Chen, Yaxin Zeng, Jinjin Kang, Minghui Hu, Nianle Li, Kun Sun, Yu Zhao

**Affiliations:** ^1^ The Eighth Affiliated Hospital, Sun Yat-sen University, Shenzhen, China; ^2^ Molecular Cancer Research Center, School of Medicine, Shenzhen Campus of Sun Yat-sen University, Sun Yat-sen University, Shenzhen, China; ^3^ Institute of Cancer Research, Shenzhen Bay Laboratory, Shenzhen, China

**Keywords:** eRNA, transcriptional regulation, chromatin looping, phase separation, epitranscriptome

## Abstract

Enhancers are a class of *cis*-regulatory elements in the genome that instruct the spatiotemporal transcriptional program. Last decade has witnessed an exploration of non-coding transcripts pervasively transcribed from active enhancers in diverse contexts, referred to as enhancer RNAs (eRNAs). Emerging evidence unequivocally suggests eRNAs are an important layer in transcriptional regulation. In this mini-review, we summarize the well-established regulatory models for eRNA actions and highlight the recent insights into the structure and chemical modifications of eRNAs underlying their functions. We also explore the potential roles of eRNAs in transcriptional condensates.

## Introduction

Enhancers are distal *cis*-regulatory elements in the genome that direct spatiotemporal transcription programs in response to diverse cues (Andersson et al., 2014; Long et al., 2016). An estimate of over 400,000 putative enhancers in human genome, plus the identification of disease-associated traits within enhancers, underscores the essence of exploring the regulatory grammar encrypted within these elements (Consortium, 2012; Long et al., 2016; Sur and Taipale, 2016; Furlong and Levine, 2018; Schoenfelder and Fraser, 2019).

The advent of state-of-art genomic approaches unveils that the human genome is pervasively transcribed, yielding a plethora of non-coding RNA (ncRNA) species (Hangauer et al., 2013). Among them, RNA transcripts emanating from enhancers, dubbed enhancer RNA (eRNAs), have attracted a particular interest considering their potential roles in enhancer regulation (De Santa et al., 2010; Kim et al., 2010; Lam et al., 2014; Li et al., 2016; Sartorelli and Lauberth, 2020; Harrison and Bose, 2022). It is noteworthy that distinct terms, e.g., eRNAs and enhancer-associated lncRNAs (elncRNAs), appear in the literature to represent transcripts from enhancer regions (Orom et al., 2010; Marques et al., 2013; Andersson et al., 2014; Li et al., 2016; Hon et al., 2017; Statello et al., 2021; Mattick et al., 2023). Strictly, eRNAs are short, bidirectional ones, which are generally non-polyadenylated and unstable, whereas elncRNAs are usually polyadenylated and have higher stability (Li et al., 2016; Statello et al., 2021; Mattick et al., 2023). However, concerning gene-activating mechanisms, elncRNAs and eRNAs share some common themes (Orom et al., 2010; Wang et al., 2011; Lai et al., 2013; Grossi et al., 2020) and we do not distinguish these different terms in this mini-review. As an integral component of active enhancers, eRNA transcription generally correlates with enhancer activation and can serve as an independent marker of active enhancers (Carullo et al., 2020). Although there isn’t yet a consensus regarding whether the functions come from the transcription process or eRNA transcripts *per se*, accumulating evidence has shown a subset of eRNAs are pivotal for the transcription of cognate targets and coined several well-appreciated themes for eRNA actions.

In this mini-review, we outline current models for eRNA actions in transcriptional regulation. In addition, we highlight recent findings concerning eRNAs secondary structure and post-transcriptional modifications in bestowing diverse functional features of eRNAs. Finally, we discuss an emerging paradigm of transcriptional condensates wherein eRNAs partaken and contribute.

### The interplay between eRNAs and protein partners in transcriptional regulation

**FIGURE 1:**
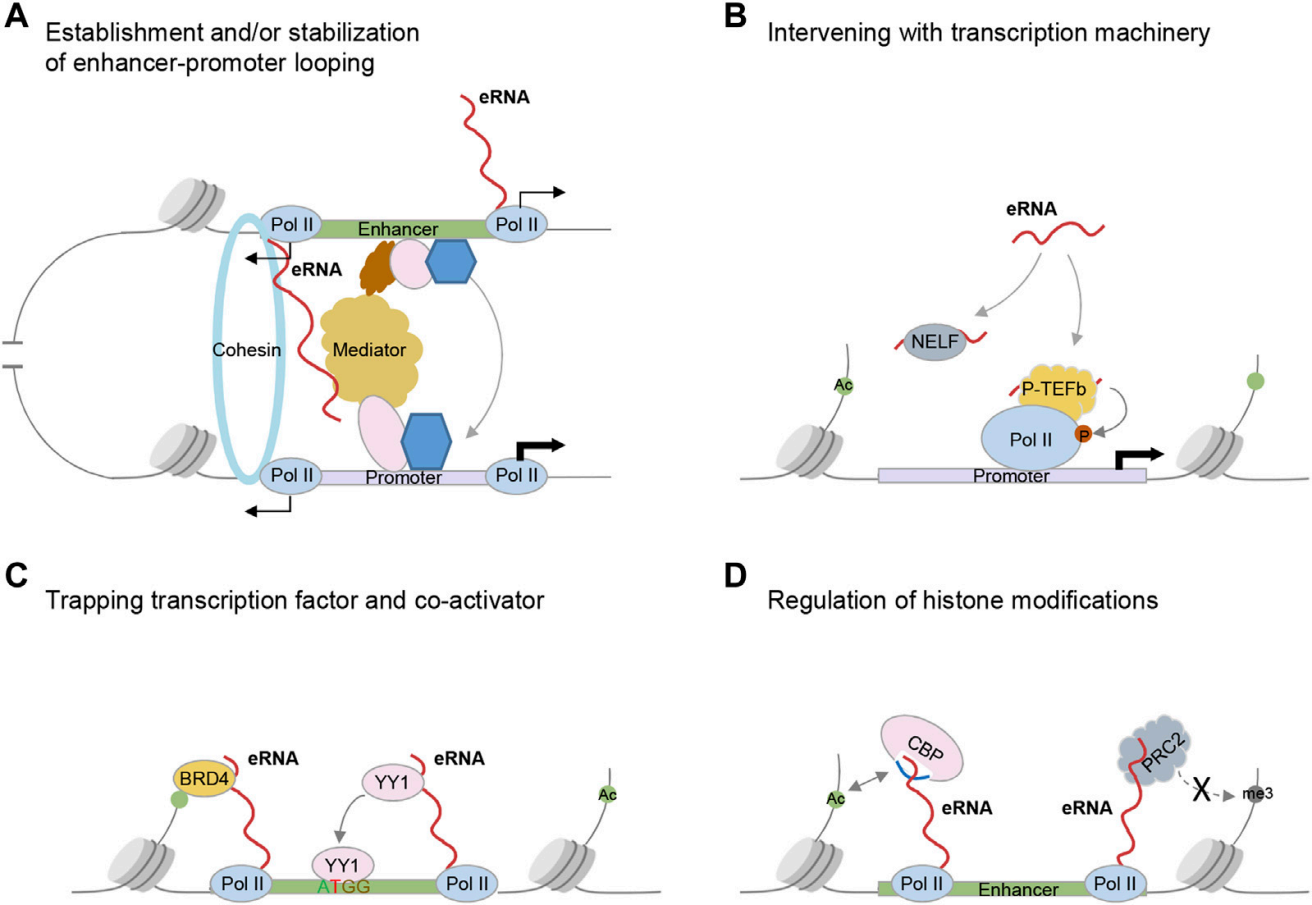
Established mechanisms underlying eRNA functions in transcriptional regulation. **(A)** Regulating chromatin looping. eRNAs interact with Cohesin complex or Mediator to establish and/or stabilize enhancer-promoter looping. **(B)** Intervening with the transcription machinery. eRNAs promote RNAP II pause release into productive elongation stage via acting as decoy for NELF and interacting with the P-TEFb. eRNAs also stimulate transcription through the intermediate hnRNPL. **(C)** Trapping transcription factors or transcription coactivators. eRNAs enhance the enhancer binding of TF YY1 and transcription coactivator BRD4 through direct interaction with them. **(D)** Modulating enhancer chromatin environment. eRNAs interact with CBP, stimulate its catalytic activity, and increase the deposition of histone acetylation on enhancers. eRNAs also inhibit the catalytic activity of PRC2 by binding the EZH2 subunit and inhibit repressive H3K27me3 deposition.

The well-appreciated model for enhancer action is that chromatin loops form between enhancers and cognate promoters bringing these two elements into physically close proximity, which involves the participation of cohesin complex, and transcriptional coactivator Mediator complex (Kagey et al., 2010). Li et al. provided the first piece of evidence that estrogen-induced eRNAs bind with SMC3 and RAD21, components of cohesin complex. Depletion of eRNAs abrogates cohesin increment to enhancers, thus abolishing enhancer-promoter interactions and target genes activation (Li et al., 2013). Similarly, Lai et al. (2013) revealed that ncRNA-a interacts with Mediator subunits and is involved in chromatin looping between ncRNA-a loci and their regulated promoters. Since then, further studies identify the direct interactions between eRNAs and chromatin looping factors [e.g., hnRNPU (Jiao et al., 2018), CTCF (Xiang et al., 2014), MED1 (Hsieh et al., 2014), MED12 (Tan et al., 2019)], suggesting modulating chromatin looping is one common theme underlying eRNA functions (Figure 1A).

In addition to regulating chromatin looping, eRNAs can directly intervene with transcription machinery (Figure 1B). Lines of evidence suggest eRNAs can modulate RNA polymerase II (Pol II) pause release (Schaukowitch et al., 2014; Zhao et al., 2016; Shi et al., 2018). Schaukowitch et al. found eRNAs bind to NELF-E, and decoy this negative elongation factor (NELF) complex away from immediate early genes, thus promoting Pol II pause release into the productive elongation stage (Schaukowitch et al., 2014). In another study, Zhao et al. uncovered that *PSA* eRNA stimulates transcription through forming a complex with the positive elongation factor (P-TEFb) (Zhao et al., 2016). Congruent with these works, the following studies added more examples demonstrating interactions between eRNAs and NELF or P-TEFb (Shi et al., 2018). Besides these direct interactions, our group identified eRNAs interact with hnRNPL via a CAAA tract and modulate the appropriate loading of hnRNPL to the target locus (Zhao et al., 2019). hnRNPL has been shown to interact with KMT3A to regulate H3K36me3 enrichment (Yuan et al., 2009) and impinge on transcription elongation via interacting with P-TEFb components, CDK9 and CCNT1 (Giraud et al., 2014). In this scenario, hnRNPL acts as an intermediate to bridge the interaction between eRNAs and transcription machinery.

Another important paradigm of eRNA functions is that eRNAs can trap transcription factors and transcription co-activators, and enhance their binding to local chromatin (Figure 1C). Sigova et al. showed nascent RNAs transcribed from enhancers and promoters, through interactions with transcription factor (TF) YY1, increase YY1 binding to these regulatory elements (Sigova et al., 2015). One recent study reinforces this idea, showing a broad scope of TFs bind to RNA through arginine-rich motif (ARM)-like domains and such interactions contribute to TF association with chromatin (Oksuz et al., 2022). Deletion of ARM-like domains skews TF nuclear dynamics: it reduces the immobile and subdiffusive fractions of TFs while enhancing the diffusing molecules. A positive-feedback loop is thus proposed that nascent RNA produced from enhancer (eRNA) or promoter regions can trap dissociating TFs through RNA-mediated weak interactions, which facilitates TFs rebind to these regulatory elements and augments the transcription outputs. Similarly, eRNAs interact directly with BRD4 via its bromodomains and promote BRD4 binding to acetylated histones, which in turn maintains enhancers in an active state (Rahnamoun et al., 2018).

Lastly, eRNAs can modulate chromatin state. Depletion of eRNAs has been shown to decrease chromatin accessibility at enhancers and cognate promoters (Mousavi et al., 2013; Tsai et al., 2018). Besides these, eRNAs can directly interact with chromatin modifiers that deposit histone acetylation or methylation marks (Figure 1D). Specifically, Bose et al. demonstrated eRNAs interact with histone acetyltransferase CBP via its RNA binding region within the activation loop of HAT domain (Bose et al., 2017). Such interaction displaces the activation loop from the catalytic site and enhances CBP binding to its histone substrate. Similarly, eRNAs can also stimulate p300 catalytic activity and increase H3K27 acetylation at enhancers (Hou and Kraus, 2022). In addition to promoting histone acetylation, eRNAs also repel the PRC2-mediated deposition of the repressive histone modification H3K27me3 (Ounzain et al., 2015). Consistently, PRC2 binds to nascent RNA promiscuously at nearly all active genes, which antagonizes its binding to chromatin and thus alleviates the deposition of the repressive H3K27me3 mark (Beltran et al., 2016; Wang et al., 2017).

As mentioned above, caution needs to be taken to discern whether eRNAs function in a transcript-dependent or -independent manner. Engreitz et al. (2016) provided compelling evidence to show regulatory roles of many lncRNA loci stem from DNA elements or transcription processes, instead of their specific transcripts. Similar findings have been reported in other works (Kaikkonen et al., 2013; Anderson et al., 2016; Paralkar et al., 2016; Winkler et al., 2022). Thus, more rigorous methodologies are warranted in future studies to distinguish this point (Engreitz et al., 2016; Joung et al., 2017).

### eRNA structures instruct their regulatory roles

Despite the substantial advances concerning eRNA functions and mechanisms, their regulatory roles instructed by eRNA structures are poorly studied. As mentioned above, eRNA can interact with and activate P-TEFb. Such interaction requires a TAR RNA-like (TAR-L) motif, whose secondary structure is akin to the 3′ end of the small nuclear RNA *7SK*. *AR-eRNA*, through competitive binding with P-TEFb, can help release P-TEFb from the inhibitory complex (7SK snRNP) and promotes effective transcription elongation (Zhao et al., 2016). On the contrary, interactions between eRNAs and NELF may not depend on structural motifs. Instead, adequate length (>200 nt) and the presence of unpaired guanosines are indispensable, which enables simultaneous and allosteric interactions between eRNAs and NELF subunits -A and -E (Gorbovytska et al., 2022).

The ^*DRR*^ *eRNA* (also known as *MUNC*) is a well-studied pro-myogenic eRNA, which is transcribed from an enhancer region of the myogenic master TF, MyoD (Mousavi et al., 2013; Mueller et al., 2015). ^*DRR*^ *eRNA* functions *in trans* to activate *Myogenin* transcription through directing cohesin loading at *Myogenin* locus (Cichewicz et al., 2018; Tsai et al., 2018). A recent study employed SHAPE-MaP (2′-hydroxyl acylation analyzed by primer extension coupled with mutational profiling) chemical probing approach to decode the secondary structure of ^*DRR*^ *eRNA* and unraveled multiple structural domains that confer distinct features of ^*DRR*^ *eRNA* for cohesion binding, genomic interaction, and gene expression regulation (Przanowska et al., 2022).

**FIGURE 2:**
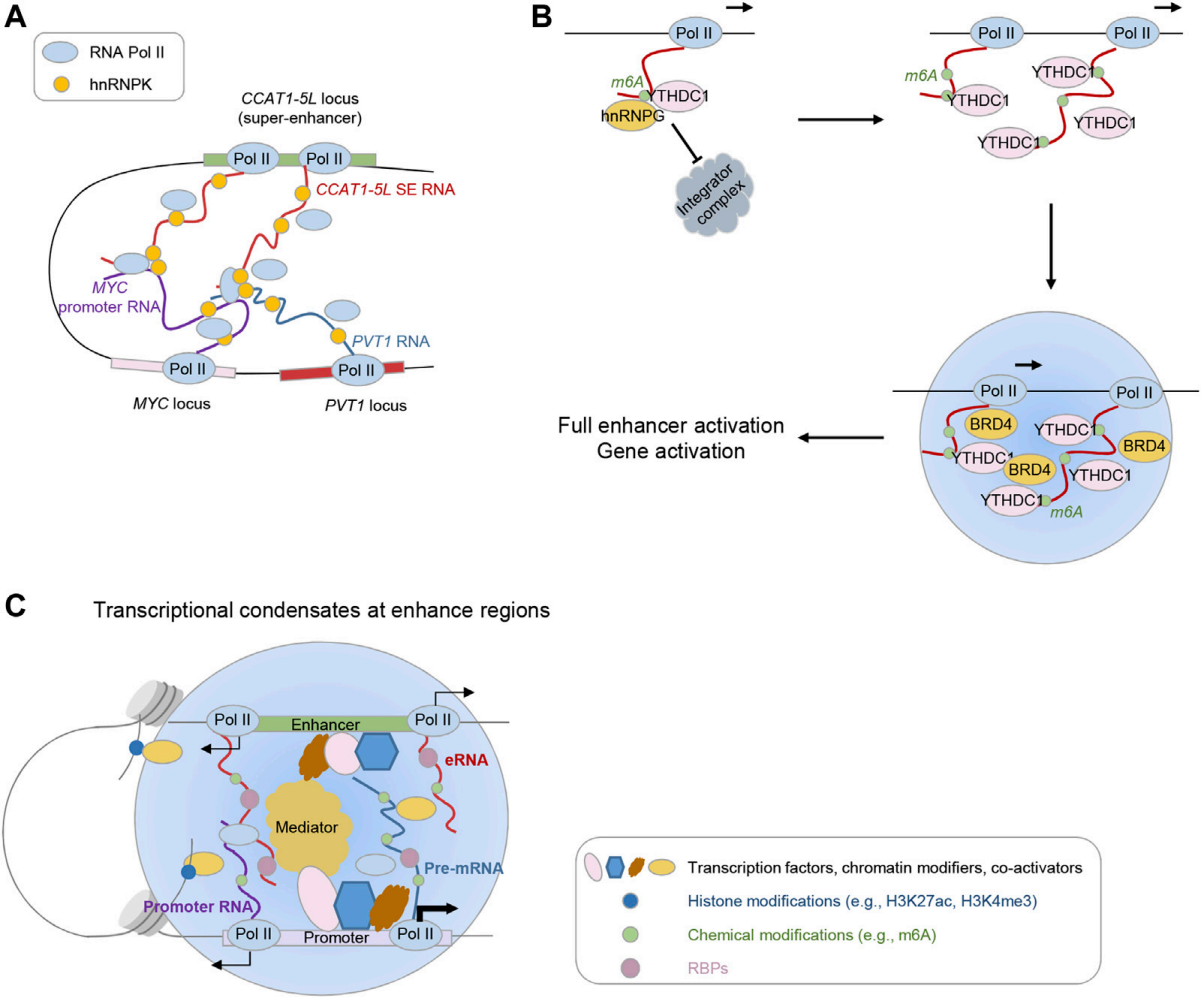
Expanding themes contributing to eRNA function. **(A)** Inter-molecular eRNA-uaRNA pair modulates enhancer-promoter chromatin interactions. **(B)** m6A modifications on eRNAs feed back on transcription. **(C)** eRNA-mediated regulation in transcriptional condensate formation.

In addition to structured features embedded in eRNAs themselves, accumulating evidence underpins the regulatory code underlying intermolecular interactions. A prominent example comes from *MALAT1*, which interacts with many pre-mRNAs at active gene loci indirectly through RNA binding protein (RBP) intermediates (Engreitz et al., 2014; West et al., 2014). Recently, Cai et al. (2020) developed a novel approach termed RIC-seq (RNA *in situ* conformation sequencing), which can map RNA-RNA interactions *in situ* in an unbiased manner, and discovered *MALAT1* interaction with highly transcribed nascent RNAs. Similarly, in this study, researchers also revealed extensive interactions between eRNAs and promoter upstream antisense RNAs (uaRNAs), which can be leveraged to infer enhancer-promoter connections. Intriguingly, modulating such interaction between eRNAs and uaRNAs influences chromatin looping (Figure 2A). Specifically, depletion of the super-enhancer-derived lncRNA *CCAT1-5L* markedly attenuates the chromatin looping between its parental *CCAT1* locus and *MYC* locus, and weakens Pol II deposition at *MYC* promoter. In this specific scenario, the interaction relies on the RBP hnRNPK, which can physically interact with Pol II and form a homodimer. Thus, hnRNPK-mediated interaction between eRNA-uaRNA pairs may serve as modulator for enhancer-promoter chromatin interactions and Pol II delivery from enhancer regions to target promoter regions.

Besides the RNA structures through RNA-RNA interactions, eRNAs can form DNA/RNA hybrid structure co-transcriptionally, termed R-loops. Competing evidence about R-loop functions comes from individual studies. Watts et al. (2022) found the enhancer RNA *AANCR* transcription leads to R-loops formation and in the R-loops eRNA is enzymatically modified to bear abasic sites, which helps stabilize R-loops, thus resulting in RNA Pol II pausing. Upon hypertonic stress, the R-loops are resolved and eRNA is fully transcribed to activate the target *APOE* activation. On the contrary, Tan-Wong et al. (2019) demonstrate that R-loops, often found at promoters, enhancers, and terminators, promote antisense transcription in these regions. More recently, local R-loops formation between an antisense eRNA *PEARL* and HS5-1 enhancer region facilitates chromatin looping between distal enhancers and target promoters (Zhou et al., 2021).

### Chemical modifications on eRNAs feed back on transcription

N6-methyladenosine (m6A) methylation, the most abundant RNA internal modification, has been shown to deposit on chromatin associated RNAs, including eRNAs (Louloupi et al., 2018; Xiao S. et al., 2019; Liu et al., 2020; Xu et al., 2021). Notably, the distribution of m6A methylation on these transcripts is not restricted to the 3′ end and is proven to regulate chromatin state and transcription directly (Louloupi et al., 2018; Xiao S. et al., 2019; Liu et al., 2020; Liu et al., 2021; Xu et al., 2021). Liu et al. found m6A-marked eRNAs, recognized by the nuclear reader YTHDC1, are subject to subsequent nuclear degradation by the nuclear exosome targeting (NEXT) complex. Knockout of the m6A writer *Mettl3* increases carRNAs abundance and promotes downstream transcription in mouse embryonic stem cells (mESCs). Mechanistically, m6A erasure upon *Mettl3* knockout stabilizes the carRNAs, rendering the following recruitment of active TFs (e.g., YY1 and CBP/EP300) and repelling of repressive factors (e.g., PRC2), thus tunes the nearby active chromatin state and stimulates downstream transcription.

The effects of m6A methylation on nuclear nascent transcripts and the transcription process could vary depending on different cell contexts. The recent two findings, on the contrary, show that m6A modification protects eRNAs from nuclear degradation, enhances the recruitment of m6A machinery components on enhancers and promoters, and stimulates effective transcription progress (Figure 2B) (Lee et al., 2021; Xu et al., 2022). In one study, Xu et al. (2022) revealed the chromatin binding of m6A methyltransferase complex (MTC) components METTL3/METTL14/WTAP locates at active enhancers and in turn decorates m6A modification on the 5′ end of nascent RNAs, neighboring to MTC chromatin binding sites. METTL3 depletion results in a loss of nascent RNAs emanating from enhancers at the TSS (transcription start site) proximal regions. Mechanistically, m6A modification recruits m6A reader/binder proteins such as hnRNPG and YTHDC1 to the nascent RNAs (including eRNAs), which protects these transcripts from cleavage by the Integrator complex. Loss of MTC would otherwise promote the recruitment of INS11 (the endonuclease subunit of the Integrator complex), leading to premature transcription termination. Of note, MTC recruitment to the promoter is augmented by active transcription elongation (Akhtar et al., 2021). Thus, m6A modification along with m6A reader proteins shields nascent eRNAs from premature termination, and the productive elongation in turn fosters MTC recruitment, establishing a positive feedback control over the transcription process.

In the other study, Lee et al. (2021) employed a high-sensitive method dubbed methylation-inscribed nascent transcripts sequencing (MINT-seq) to capture m6A methylome directly on nascent RNAs. They uncovered m6A is pervasively decorated with enrichment in the middle of eRNA transcripts and m6A modification positively correlates with eRNA length and abundance. In agreement with the canonical “RRACH” motif identified on mRNAs, “GGACT” motif sequences are identified with eRNA m6A peaks. Functionally, m6A-modified eRNAs can stimulate enhancer activation through reader protein YTHDC1 recruitment. Targeted m6A erasure, genetic and chemical perturbation of m6A writer and reader impair the enhancer activation, eRNA transcription, and subsequent target gene activation. Mechanistically, YTHDC1 can phase-separate into liquid-like condensates and co-assemble into BRD4 transcriptional condensates, while m6A-eRNAs presence augments the size of condensates. Concordantly, either perturbation of YTHDC1 levels or its condensate formation ability attenuates BRD4 recruitment to enhancers and BRD4 condensate formation.

In addition to m6A modification, enrichment of 5-methylcytosine (m5C) marked eRNAs were found at a set of enhancers upon metabolic stress (Aguilo et al., 2016). Under this circumstance, the interaction between PGC-1a and the NOP2/Sun RNA methyltransferase 7 (NSUN7) is essential in instructing m5C deposition on eRNAs.

### eRNAs and transcriptional condensates

Recent studies have shown liquid-liquid phase separation (LLPS) occurs at super-enhancers, which compartmentalizes crowded transcription regulators (e.g., TFs, transcriptional co-activators, RNA Pol II, and RNA) and promotes the formation of transcriptional condensates (Hnisz et al., 2017; Boija et al., 2018; Cho et al., 2018; Sabari et al., 2018; Shrinivas et al., 2019). Considering the established multivalent interactions between eRNAs with a myriad of factors (e.g., TFs, chromatin modifiers, DNA, RNA), eRNAs could potentially play a broad role in the formation of transcriptional condensates at enhancers (Roden and Gladfelter, 2021). Nair et al. (2019) recently reported an indispensable role of eRNA in controlling the assembly of MegaTrans complex at the ligand-activated enhancers, which exhibit properties of phase-separated components. Intriguingly, the complex components include several transcription factors (e.g., GATA3, ERα, RARA, FOXA1, AP2γ), which harbor intrinsically disordered regions (IDRs). The authors demonstrated two of them, GATA3 and ERα, are capable of liquid phase condensation at enhancers. Depletion of eRNA affects the diffusion properties of MegaTrans components, thus abolishing the full assembly of MegaTrans at the cognate enhancer. Notably, chronic enhancer activation alters the physicochemical properties of this enhancer RNA-dependent ribonucleoprotein (eRNP) complex to a more gel-like state. This study provides compelling evidence showing eRNAs directly contribute to the formation of phase-separated condensates and enhancer activation.

Based on current findings, we can extrapolate eRNAs play a broad role in controlling the formation, dissociation, and dynamics of transcriptional condensates at enhancers and/or cognate promoters via scaffolding multivalent interactions between condensate components (Maharana et al., 2018; Henninger et al., 2021; Quinodoz et al., 2021; Roden and Gladfelter, 2021). First, eRNAs may have a role in contributing to the formation of transcriptional condensates. Many eRNAs-interacting protein partners, as mentioned above, harbor IDRs that are essential in the induction of phase separation. For example, eRNAs interact with MED1 and BRD4, the IDRs of which have been demonstrated to foster super-enhancer formation through phase separation (Cho et al., 2018; Sabari et al., 2018). eRNAs also interact with P-TEFb and the recent finding supports the promoting role of CCNT1, a component of P-TEFb, in phase separation via its histidine-rich domain, which subsequently compartmentalizes RNA Pol II C-terminal domain (CTD) into CCNT1 droplets to ensure CTD hyperphosphorylation and transcription elongation (Lu et al., 2018). In addition to TFs and co-activators, increasing evidence has shown RBPs pervasively bind to regulatory elements and mediate the phase separation (Xiao et al., 2019a; Shao et al., 2022). Shao et al. discovered one RBP PSPC1 exhibits liquid-like properties and the presence of RNA augments the PSPC1-mediated transcriptional condensates that compartmentalize the CTD for enhanced phosphorylation. The low-complexity sequences (LCS) and RNA recognition motifs (RRMs) of PSPC1 are the prerequisites for the synergistic interplay between PSPC1 and RNA, the resultant PSPC1 chromatin binding and phase separation. Remarkably, the discovery that chrRBPs tend to co-occupy at regulatory regions, such as super-enhancers and promoters, provides a chance that diverse RBPs act collaboratively, in synergy with RNAs from these regulatory elements, in promoting the formation of transcriptional condensates. Second, eRNAs may not only engage in the formation but also regulate the dissociation and composition of transcriptional condensates. Maharana et al. proposed that RNA concentration determines distinct phase separation behaviors: higher RNA concentration impedes phase separation of RBPs in the nucleus, while lower RNA concentration facilitates aggregation (Maharana et al., 2018). Consistently, Henninger et al. (2021) recently reported low levels of RNA generated due to transcription initiation at regulatory elements, including eRNAs, promote condensate formation, whereas high production of RNAs during transcription elongation results in condensate dissolution. Considering the majority of eRNAs are short, unstable, and lowly expressed, eRNAs more likely partaken in the formation of transcriptional condensates. Interestingly, a recent work revealed nascent RNAs primarily impede the association of diverse categories of proteins with chromatin, including transcriptional regulators and chromatin modifiers (Skalska et al., 2021). RNA directly binds to these factors, and in turn blocks their binding to nucleosomes, suggesting an antagonistic relationship between their RNA- and chromosome-binding. Whether these proteins contribute to the formation of phase-separated condensates awaits further investigation. In addition, the phosphorylation status of RNAPII CTD affects the compartmentalization of RNAPII into distinct condensates (Guo et al., 2019). As eRNAs are proven to interact with protein components from different condensates (e.g., Mediator complex and RBPs involved in RNA processing and splicing), they are likely to influence the dynamic exchange of RNAPII between different condensates.

## Conclusion

Despite the substantial progress of eRNA studies, much more efforts are warranted in delineating their functions and underlying mechanisms, considering the heterogenous nature in terms of their expression, length, secondary structures, and post-transcriptional modifications. For instance, our understanding of eRNA structures is poorly explored. To tackle this situation, more structural studies (e.g., SHAPE-MaP) are anticipated to uncover intramolecular secondary structures crucial for distinct properties of eRNAs. Equally important is the cataloging a more comprehensive list of eRNA binding partners (e.g., RBPs). Analyses of such data can provide insights into how eRNAs interact, and whether common sequence motifs or structural features of eRNAs exist conferring the interaction specificity. It will be also important to further explore the recently identified intermolecular RNA-RNA interactions and RNA-chromatin interactions, which present an intriguing possibility that eRNAs potentially participate in nuclear compartmentalization (Cai et al., 2020; Quinodoz et al., 2021). Besides, the existence and functions of epitranscriptomic modifications on eRNAs, such as m6A, m5C, hydroxymethyl cytosine (5hmC), and methyl-1-adenosine (m1A), need to be further explored. One trending direction is to demystify the regulatory feedback from these chemical modifications on nascent RNAs (including eRNAs) to chromatin and transcription. As mentioned above, pieces of evidence point to the involvement of eRNAs in phase-separated transcriptional condensates. Multivalent interactions mediated by eRNAs, e.g., RNA-RNA, RNA-protein, RNA-DNA interactions, render them great potential in mediating phase separation (Figure 2C). Several important questions need to be addressed in the future. How do eRNAs contribute to the formation of transcriptional condensates and what features are important (e.g., length, motifs, secondary structures, intermolecular RNA-RNA interactions, and post-transcriptional modifications)? Do eRNAs regulate the transition from transcription initiation into elongation condensates? In addition, the linkage is largely unclear between eRNA-involved transcriptional condensate formation and higher-order 3D genome organization. Finally, functional investigations are required to delineate the roles of eRNAs in disease entities (Zhang et al., 2019; Chen and Liang, 2020) and to dissect how the altered eRNA features favor disease development. Answers to these questions will provide deeper insights not only into eRNA functions and regulatory mechanisms but also into eRNA-centric therapeutic strategies.
